# Exogenous hydrogen sulfide mitigates the fatty liver in obese mice through improving lipid metabolism and antioxidant potential

**DOI:** 10.1186/s13618-014-0022-y

**Published:** 2015-01-10

**Authors:** Dongdong Wu, Nairui Zheng, Kunqing Qi, Huijun Cheng, Ziqiang Sun, Biao Gao, Youjing Zhang, Wuyan Pang, Chaoshen Huangfu, Shaoping Ji, Mengzhou Xue, Ailing Ji, Yanzhang Li

**Affiliations:** Medical College of Henan University, Kaifeng, 475004 Henan China; Institute of Environmental Medicine of Henan University, Kaifeng, 475004 China; Nursing College of Henan University, Kaifeng, 475004 China; Department of Neurology, the First Affiliated Hospital, Institute of Neurological Disorder, Henan University, Kaifeng, 475004 China; Huaihe Hospital of Henan University, Kaifeng, 475004 China

**Keywords:** Hydrogen sulfide, Fatty liver, Mitigation, Lipid metabolism, Antioxidant

## Abstract

**Background:**

Nonalcoholic fatty liver disease (NAFLD) is the most common liver disease in the world. Hydrogen sulfide (H2S) plays an important role in physiology and pathophysiology of liver. However, whether exogenous H2S could mitigate the hepatic steatosis in mice remains unclear. The aim of this study is to evaluate the effects of H2S on fatty liver.

**Methods:**

C57BL/6 mice were fed with either a high-fat diet (HFD) or a normal fat diet (NFD) for 16 weeks. After 12 weeks of feeding, the HFD-fed mice were injected one time per day with NaHS or saline for the followed 4 weeks.

**Results:**

Compared to NFD, HFD could induce an accumulation of lipids in liver and a damage of hepatic structure. Compared to saline treatment, in the liver of HFD fed mice H2S treatment could significantly (1) recover the structure; (2) decrease the accumulation of lipids including triglyceride (TG) and total cholesterol (TC); (3) decrease the expression of fatty acid synthase (FAS) and increase the expression of carnitine palmitoyltransferase-1 (CPT-1); (4) reduce malondialdehyde (MDA) levels; (5) increase the activities of superoxide dismutase (SOD) and glutathione peroxidase (GPx).

**Conclusion:**

H2S could mitigate the fatty liver by improving lipid metabolism and antioxidant potential in HFD-induced obese mice.

## Introduction

Nonalcoholic fatty liver disease (NAFLD) is the most common liver disease in the world, which is caused by abnormal accumulation of triglyceride inside the hepatocytes [1]. NAFLD may progress to non-alcoholic steatohepatitis (NASH), fibrosis, cirrhosis, and hepatocellular cancer, and is also a risk factor for the development of type 2 diabetes and cardiovascular disease (CVD) [2].

Hydrogen sulfide (H2S) is an endogenous signaling molecule in mammalians [3], which is synthesized enzymatically by cystathionine β-synthase (CBS), cystathionine γ-lyase (CSE), and 3-mercaptopyruvate sulfurtransferase (MST) during cysteine metabolism [3]. Accumulating evidence suggests that H2S plays an important role in physiology and pathophysiology of liver [4]. The dysregulation of endogenous H2S is correlated with the symptoms of diabetes [5,6] and liver cirrhosis [7]. Blood levels of H2S in type 2 diabetic patients (T2D) are lower than that of the controls [8]. Endogenous formation of H2S is impaired in rats with NASH and treatment with H2S can prevent NASH in rats possibly through abating oxidative stress and suppressing inflammation [9]. The administration of sodium hydrosulfide (NaHS)-a H2S donor in rodents protects against liver injury [4], which is induced by ischemia reperfusion [10], acetaminophen [11], or carbon tetrachloride [7]. Application of H2S also exhibits the effects on mitochondrial function [12,13], anti-oxidative stress [14,15], apoptosis [16], inflammation [17], angiogenesis [18–20], and blood pressure [21–23].

Recent studies have demonstrated that CSE, CBS, and MST proteins could be detected in the liver, and they contribute to liver production of H2S to different extents [4,9,24–26]. High fat diet can induce a down-regulation of CSE/CBS and results in less H2S production in the liver of rodents [25]. Based on this point, we propose that increasing H2S by administration of NaHS may restore liver from steatosis in HFD-induced obese mice. To prove our hypotheses, we employed a diet-induced obesity (DIO) mouse model to evaluate the effects of exogenous H2S on fatty liver of obese mice. We found that the administration of H2S donor NaHS could mitigate the fatty liver by improving lipid metabolism and antioxidant potential in HFD-induced obese mice. H2S products may be used as therapeutical drugs for the NAFLD.

## Materials and methods

### Animals

The protocols for animal experiments were reviewed and approved by the Animal Care and Use Committee of Henan University. Eight weeks old male C57BL/6 mice were purchased from Nanjing Biomedical Research Institute of Nanjing University (Nanjing, China) and housed in a temperature and humidity controlled environment on a 12 hour light/dark cycle with free access to food and water. The mice were fed either a chow (normal fat diet, NFD; KeAoXieli, Beijing, China) or a high fat diet (HFD, 45% kcal as fat, MD12032, Medicience Ltd., Jiangsu, China) for total 16 weeks. After 12 weeks of feeding, the HFD feeding mice were divided into HFD group and HFD + H2S group. Each group contained 10 HFD feeding mice. The mice from HFD group and HFD + H2S group were received an intraperitoneal (i.p.) injection of either saline (10 ml/kg/day) or NaHS (50 umole/kg/day, dissolved in saline, 10 ml/kg body weight) for 4 weeks. At the end of experiments, the mice were killed. Tissues were rapidly removed from the mice and frozen either in liquid nitrogen or embedded in frozen section compound (Surgipath FSC22, Leica) and stored at −20°C or by immersion in 4% formalin.

### Histological analysis

For histology, livers were fixed in formalin, embedded in paraffin, and cut into 5 μm thick sections. The sections of livers were stained with hematoxylin and eosin. For oil red O (ORO) staining, the frozen liver tissues were cut into 10 μm-thick sections, stained with oil red O, and counterstained with hematoxylin [27]. All sections were scanned at an absolute magnification of 200× under a light microscope (Olympus, BX51 microscope, Tokyo, Japan) and analyzed using Image-J software (NIH). The accumulation of hepatic lipid droplets was presented as the percentage of the blank area (lipid droplets, H&E staining) or the red staining area (lipid droplets, ORO staining) relative to the whole area of the photomicrograph.

### Determination of liver triglyceride (TG) and total cholesterol (TC)

Liver tissues were homogenized on ice with the sample buffer [tissue weight: buffer = 1(g): 9(ml)]. The homogenates were centrifuged at 2500 rpm for 10 min. The supernatants were transferred to the new tubes. Triglyceride (TG) and total cholesterol (TC) in the supernatants were measured by enzymatic colorimetric methods using commercial kits according to the manufacturer’s protocols (Nanjing Jiancheng Bioengineering Institute, Nanjing, China).

### RNA extraction and real-time PCR

Total RNA was isolated from liver tissues using TRIzol reagent (life technologies), treated with DNase I (Roch), and purified using an RNA Clean Up Kit (Kangwei, China). One microgram of total RNA was used for cDNA synthesis using a cDNA Reverse Transcription Kit (Kangwei, China). The primer sequences for fatty acid synthase (FAS) [28], carnitine palmitoyltransferase-1 (CPT-1) [29], and 18s rRNA [30] are shown in Table 1. PCR tests were performed in a total volume of 20 μl using the following thermal cycling parameters: 94°C for 2 min, and then 40 cycles of 94°C for 30s, 58°C for 30s, and 72°C for 1 min. The mRNA expression levels of the test genes were normalized to 18s rRNA levels.

**Table 1:** **Sequences of primers used in the study**

**Genes**	**Forward (5′-3′)**	**Reverse (5′-3′)**	**GenBank accession**
18s rRNA	AGTCGGCATCGTTTATGGTC	CGAAAGCATTTGCCAAGAAT	X00686
FAS	GGTCGTTTCTCCATTAAATTCTCAT	CTAGAAACTTTCCCAGAAATCTTCC	NM_007988
CPT-1	CCAGGCTACAGTGGGACATT	GAACTTGCCCATGTCCTTGT	AF017175

### Measurement of hepatic FAS and CPT-1 levels

A small piece of the frozen liver tissue was homogenized in PBS (pH 7.2-7.4) on ice by manual grinding. Insoluble material was removed by centrifugation for 15 min at 2000 rpm, 4°C. Hepatic FAS and CPT-1 levels were measured using ELISA kits according to the manufacturer’s protocol (Tianchi, Zhengzhou, China).

### Determination of hepatic lipid peroxidation and antioxidant enzymes

Malondialdehyde (MDA) is a product of the lipid peroxidation and frequently used as a biomarker of oxidative stress [31]. In present study, the lipid peroxidation was determined by measuring the formation of thiobarbituric acid-reactive substances (TBARS) spectrophotometrically, and expressed as malondialdehyde (MDA) concentration [32]. The production of MDA, and activities of glutathione peroxidase (GPx), catalase (CAT), total superoxide dismutase (T-SOD), Cu/Zn-SOD, and Mn-SOD in liver tissues were measured with assay kits according to the instructions of manufacturer (Nanjing Jiancheng Bioengineering Institute, Nanjing, China).

### Statistical analysis

Data were presented as means ± standard error of mean (SEM). Differences between groups were analyzed by one-way analysis of variance (ANOVA) using SPSS 17.0 software, followed by Tukey’s test. A value of P < 0.05 was considered significant.

## Results

### H2S decreases the liver weights of obese mice

**Figure 1:**
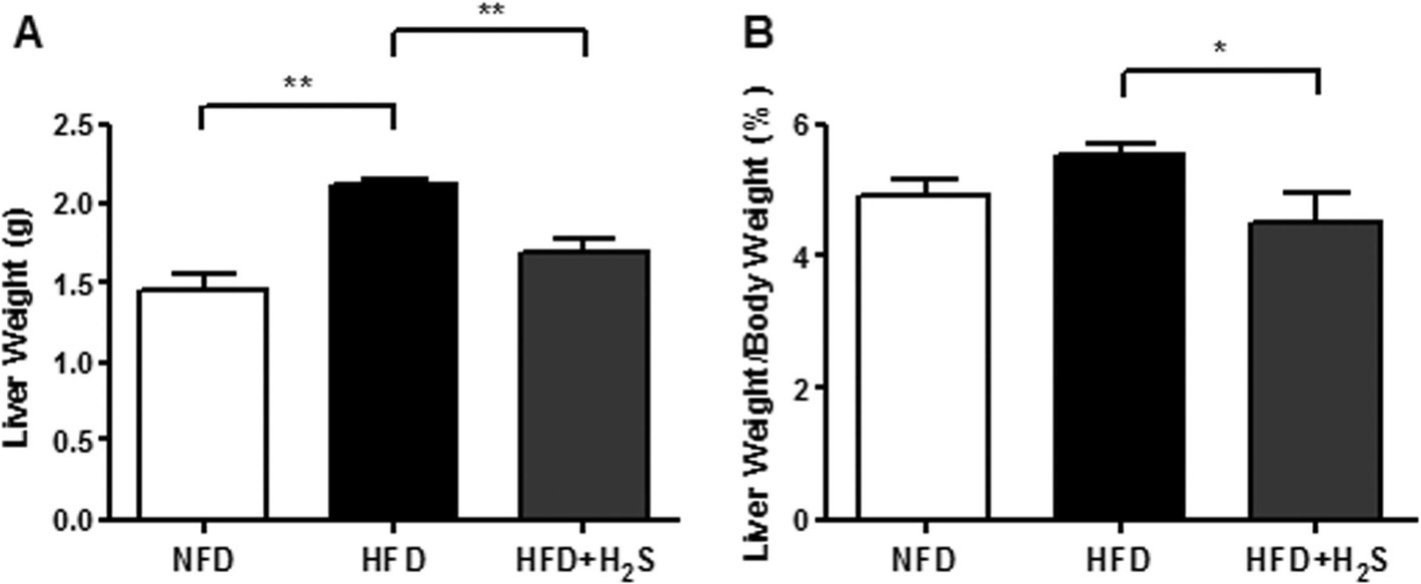
**Effects of H2S on the liver mass of HFD-induced obese mice. (A)** Liver weight. **(B)** The ratio of liver weight vs body weight. Data are means ± SEM (n = 10).*p < 0.05, **p < 0.01 vs. HFD group.

After 16 weeks feeding, the mice were killed and the wet weight of the liver was monitored. Compared to the NFD fed mice, HFD fed mice showed increased liver weight (Figure 1A, P < 0.01). H2S administration tended to significantly lower the liver weight compared to HFD group (Figure 1A, P < 0.05). HFD feeding did not affect the liver/body weight ratio (Figure 1B). H2S treatment markedly reduced the liver/body weight ratio (Figure 1B, P < 0.05).

### H2S reduces the accumulation of lipids in the liver of obese mice

**Figure 2:**
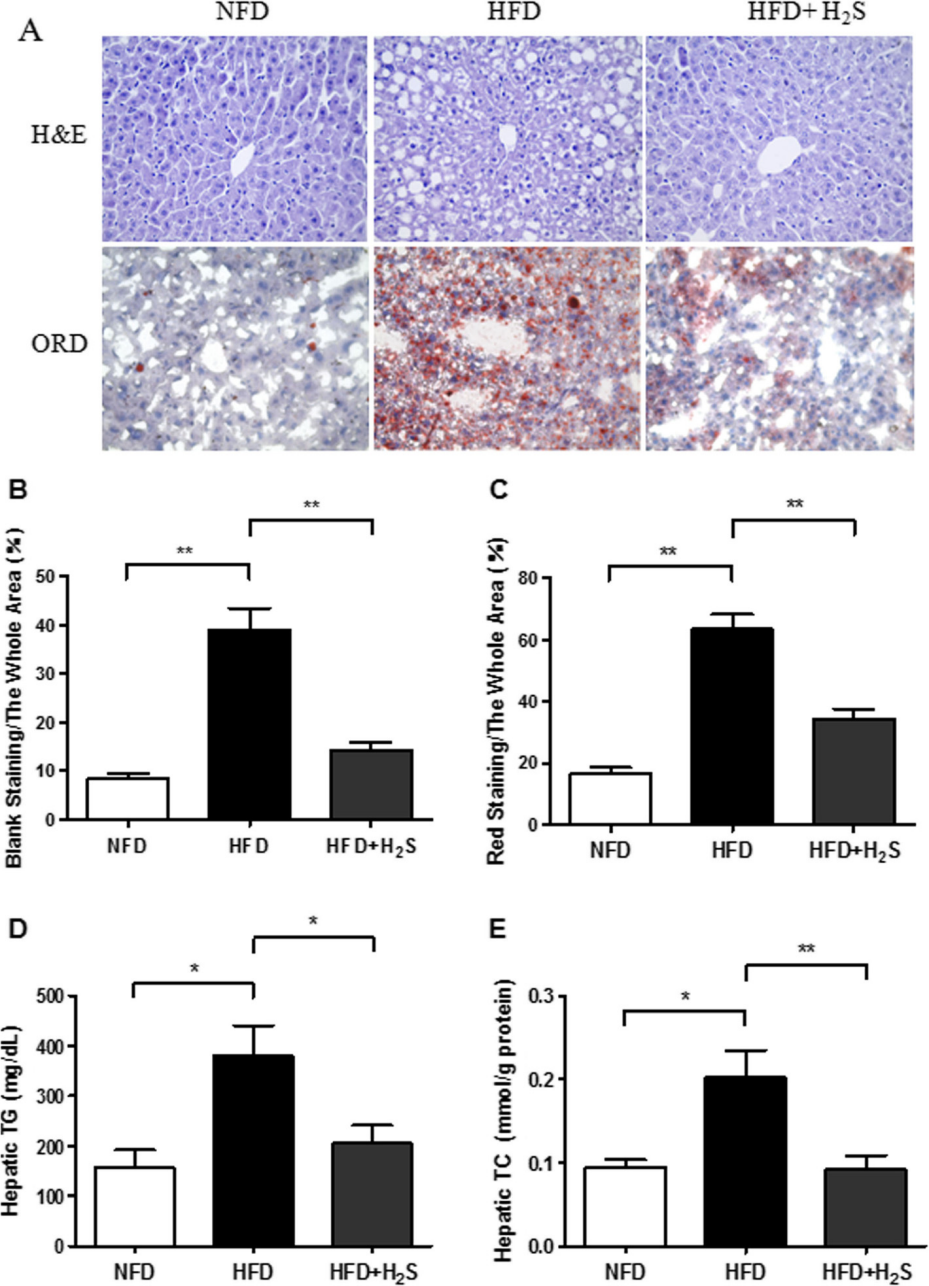
**Effects of H2S on HFD-induced hepatic steatosis in mice. (A)** Representative images of H&E and ORO staining of liver sections (200×). **(B-C)** The quantification of the hepatic lipid droplets accumulation was presented as the percentage of the blank area (lipid droplets, H&E staining) or the red staining area (lipid droplets, ORO staining) relative to the whole area of the photomicrograph. **(D-E)** Hepatic triglyceride and total cholesterol levels. Data are means ± SEM (n = 10). *p < 0.05, **p < 0.01 vs HFD group.

Representative photomicrographs of liver H&E as well as ORO staining and quantitative analysis of hepatic triglyceride content were shown in Figure 2. Compared to NFD group, HFD caused significant vacuolar degeneration of hepatocytes, disruption of normal hepatic lobules, and inflammatory cells infiltration in H&E-stained liver sections (Figure 2A). HFD-induced fat accumulation was characterized mainly by microvesicular steatosis as shown by ORO staining (Figure 2A), suggesting impaired mitochondrial function [33]. Compared to HFD group, H2S treatment significantly reduced hepatic lipid droplets and macrovesicular steatosis (Figure 2B, C, P < 0.01). Furthermore, the contents of TG and TC were analyzed to determine the extent of lipid accumulation in the liver. As shown in Figure 2D and 2E, HFD-fed mice had increased TG and TC contents in the liver compared to that of NFD group, while H2S treatment markedly reduced hepatic TG and TC contents (P < 0.05). These results suggest that H2S may restore the liver from hepatic steatosis in obese mice.

### H2S modulates the expression of hepatic FAS and CPT-1

**Figure 3:**
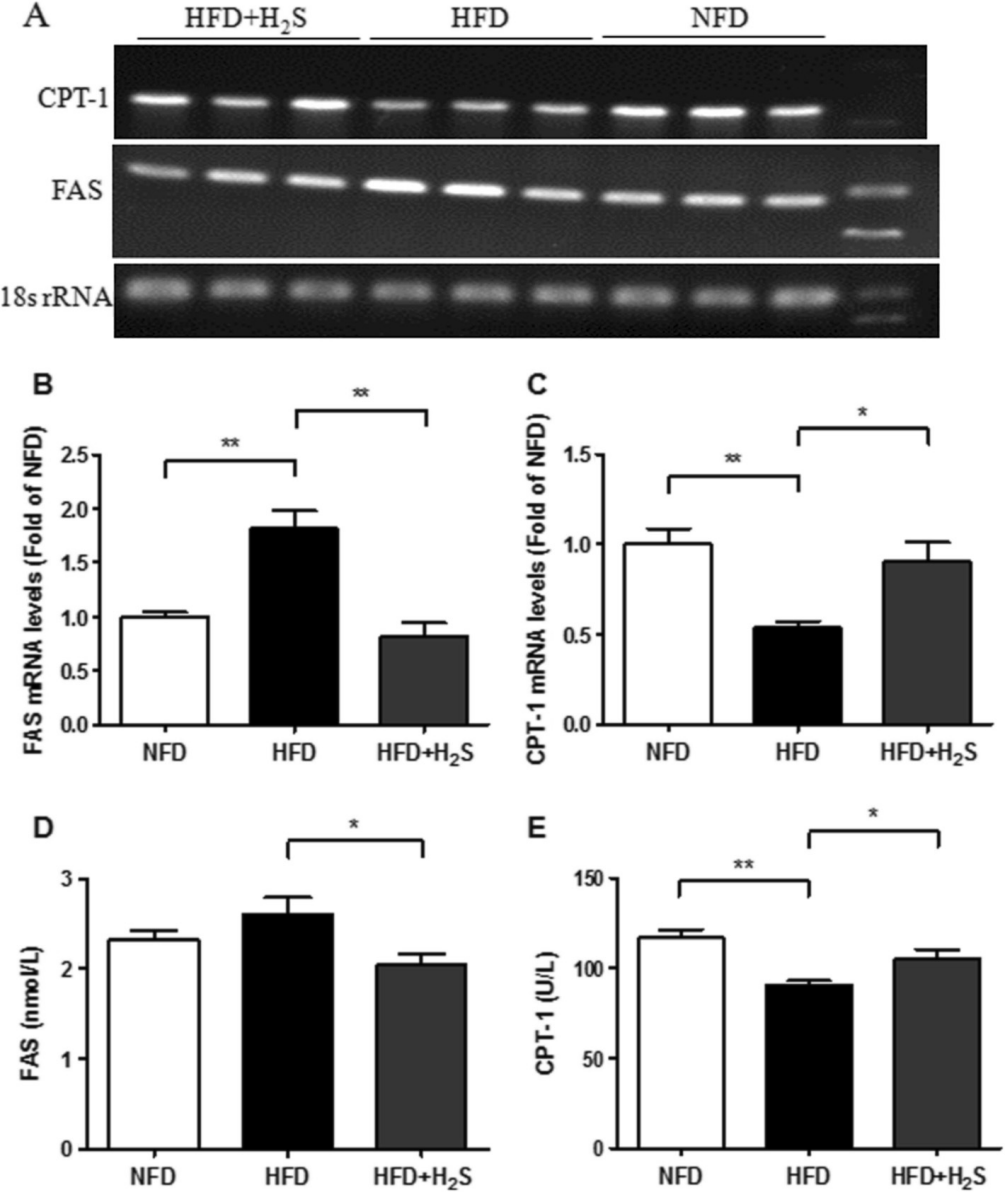
**Effects of H2S on mRNA and protein levels of FAS and CPT-1 in the liver of HFD-fed mice. (A-C)** The mRNA levels of FAS and CPT-1 were examined by RT-PCR (n = 3). **(D-E)** The protein levels of FAS and CPT-1 were examined by ELISA (n = 10). Data are means ± SEM. *p < 0.05, **p < 0.01 vs HFD group.

FAS and CPT are two representative enzymes involved in lipid biogenesis and energy expenditure [33]. The expression levels of FAS and CPT-1 were assessed to investigate whether H2S would affect lipid metabolism in the liver. Although high-fat feeding markedly increased the mRNA expression level of FAS (Figure 3A, B, P < 0.05), there was no significant change in FAS protein expression between the NFD and HFD groups (Figure 3D). Compared to HFD group, H2S treatment reduced FAS expression at both the mRNA and protein levels (Figure 3A, B, D, P < 0.05). The mRNA and protein expression levels of CPT-1 were significant lower in HFD group than that in NFD group (Figure 3A, C, E, P < 0.01), while H2S treatment increased CPT-1 expression significantly at both the mRNA and protein levels (Figure 3A, C, E, P < 0.05). Down-regulation of FAS in liver indicates that the synthesis of lipids decreased. Up-regulation of CPT-1 suggests that the expenditure of lipids increased. These results indicate that H2S reduces the accumulation of lipids in liver possibly through down-regulation of FAS and up-regulation of CPT-1.

### H2S regulates lipid peroxidation and antioxidant enzyme activities in liver

**Figure 4:**
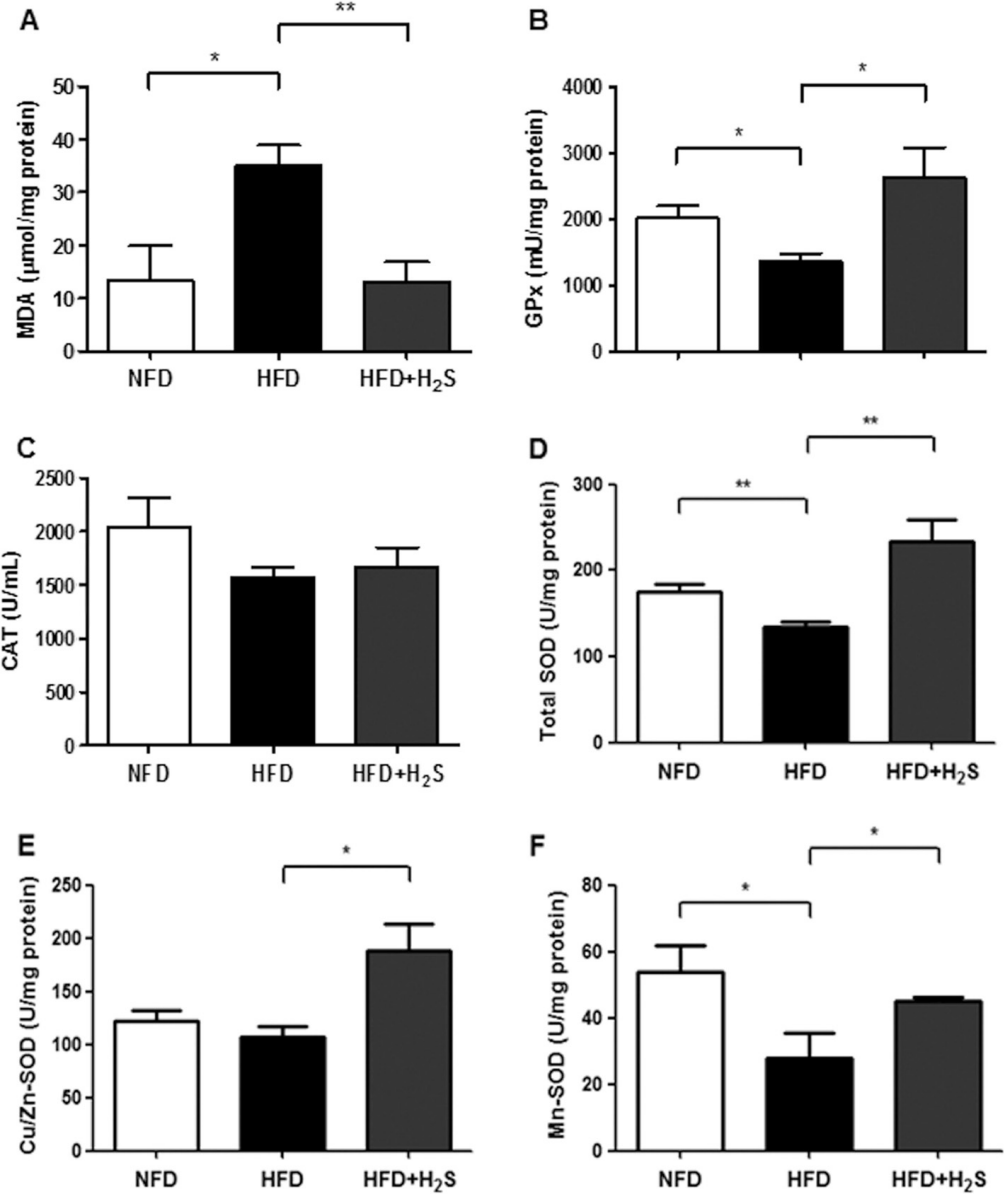
**Effects of H2S on the MDA levels and the activities of antioxidant enzymes in the liver of HFD-fed mice. (A)** MDA levels. **(B-F)** The activities of GPx, CAT, total SOD, Cu/Zn-SOD, and Mn-SOD. Data are means ± SEM (n = 10). *p < 0.05, **p < 0.01 vs HFD group.

HFD feeding significantly increased hepatic MDA formation (end-product of lipid peroxidation, as a biomarker of oxidative stress) and suppressed the activities of hepatic antioxidant enzymes including GPx, T-SOD, Cu/Zn-SOD, and Mn-SOD. H2S treatment markedly decreased hepatic MDA formation and restored the activities of hepatic GPx, T-SOD, Cu/Zn-SOD, and Mn-SOD (Figure 4A, B, D, E, F, P < 0.05). No significant differences were observed in the catalytic activity of CAT among groups, suggesting that HFD feeding and H2S treatment did not affect the activity of CAT (Figure 4C). These results suggest that H2S protects the liver injury induced by HFD feeding through reducing the oxidative stress and increasing the activities of the antioxidant enzymes in the liver of HFD fed mice.

## Discussion

**Figure 5:**
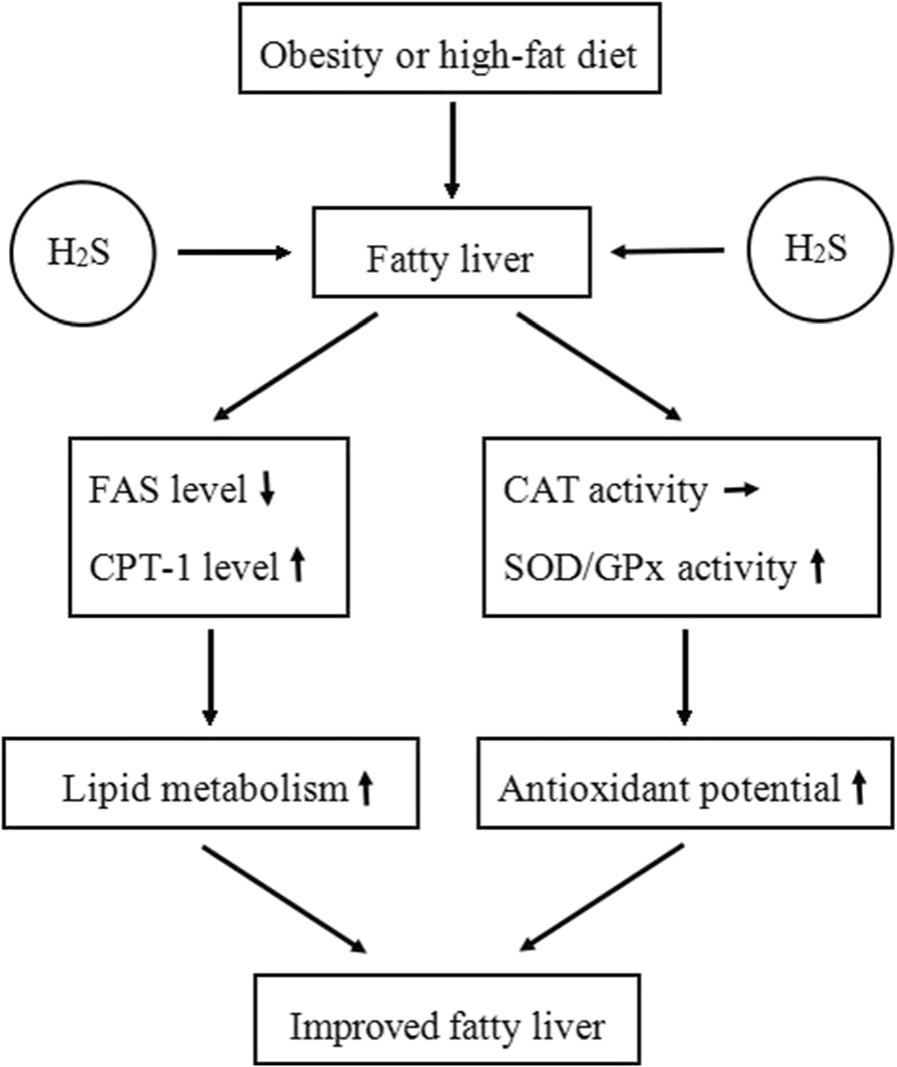
**Effects of H2S on fatty liver.** H2S mitigates fatty liver by improving lipid metabolism and antioxidant potential. H2S, hydrogen sulfide; FAS, fatty acid synthase; CPT-1, carnitine palmitoyltransferase-1; CAT, catalase; SOD, superoxide dismutase; GPx, glutathione peroxidase. ↓ shows decrease; ↑ shows increase; → shows no obvious change.

The present study was designed to determine the effects of H2S on hepatic steatosis using a mouse model of HFD-induced obesity. We found that the administration of H2S donor NaHS could (1) reduce the liver weight; (2) reduce the contents of TG and TC in hepatocytes; (3) increase CPT-1 expression and decrease FAS expression; (4) increase the activities of antioxidant enzymes such as SOD and GPx. Based on our findings, we proposed a possible mechanism of H2S in mitigating fatty liver as shown in Figure 5. H2S can restore the liver from steatosis through suppressing hepatic fat accumulation by down-regulation of FAS and up-regulation of CPT-1. Additionally, H2S mitigates the fatty liver through up-regulating the activities of antioxidant enzymes (GPx and SOD). Take together, H2S products may be used as a therapeutical drug for the NAFLD.

Previous studies have demonstrated that a dramatic increase in overall liver weight and/or liver/body weight ratio could be observed in obese mice [34,35]. In the current study, HFD feeding resulted in a significant increase in overall liver weight, but did not change the liver/body weight ratio. However, H2S administration significantly reduced both the liver weight and liver/body weight ratio. Taken together, our results demonstrate that H2S has the ability to decrease liver weight in HFD-fed mice.

As a central metabolic organ, the liver plays a vital role in various aspects of lipid and glucose metabolism in response to nutritional and hormonal signals [4,36]. Liver dysfunction could lead to dysregulation of lipid metabolism, including alterations in fatty acid biosynthesis, beta-oxidation and very low density lipoprotein (VLDL) secretion, which subsequently influences serum levels of cholesterol and lipoproteins [4,37,38]. In the present study, our observation proved obvious fat accumulation in the liver after feeding of mice with 45% HFD for 16 weeks, which was evidenced by H&E and ORO staining (Figure 2). These phenotypic alterations were clearly abolished by H2S treatment for 4 weeks. In addition, H2S administration significantly reduced the hepatic triglyceride and cholesterol levels in HFD-induced obese mice, which was similar to a previous study that it inhibited cholesterol and triglyceride synthesis in liver of MCD diet-fed rats [9].

FAS and CPT-1 are two representative enzymes which play important roles in lipid biogenesis and energy expenditure [33]. FAS could effectively convert acetyl-CoA and malonyl-CoA into palmitate in a process that requires NADPH, which is supplied by both the pyruvate cycle and the pentose phosphate pathway [39]. CPT-1, an enzyme that resides in the outer mitochondrial membrane, is responsible for facilitating the entry of fatty acids into the mitochondria for oxidation [40,41]. In this study, H2S treatment markedly reduced FAS expression at both the mRNA and protein levels compared to the HFD group. In contrast, the increased expression of CPT-1 was observed in HFD + H2S group. These results suggest that H2S might prevent the development of fatty liver by modulating the expression of hepatic lipid-regulating enzymes.

Reactive oxygen species (ROS) are free radicals derived from oxygen and non-radical species such as hydrogen peroxide (H2O2) [42]. Under normal physiological conditions, the levels of ROS generated from the oxidative processes within the cell can be effectively reduced by the antioxidant defense systems, which represent a number of enzymes such as SOD, CAT, and GPx, as well as non-enzymes include glutathione (GSH), vitamin E, vitamin A, and vitamin C [43,44]. Recent studies have indicated that ROS plays an important role in various liver diseases as a significant causative factor, including NAFLD/NASH [43,45,46]. Oxidative stress is defined as an imbalance between the formation of ROS and the antioxidant defense capacity [47]. A central measurement of oxidative stress is lipid peroxidation (LPO), as indicated by MDA levels, which can contribute to a consequence of cellular damage [44]. It has been reported that H2S could prevent the elevation of MDA induced by MCD feeding [9]. Our results showed that H2S treatment significantly decreased hepatic MDA formation in HFD group. On the other hand, our present study found HFD diet declined hepatic anti-oxidative defense via down-regulating the activities of GPx and SOD. H2S effectively restored the activities of these enzymes. These findings indicate that H2S could provide anti-oxidative effects against HFD-induced hepatotoxicity.

Based on the results obtained in the present study, it is concluded that administration of H2S could mitigate the fatty liver by suppressing hepatic fat accumulation through down-regulation of FAS and up-regulation of CPT-1 in HFD-fed obese mice. In addition, H2S may suppress oxidative stress by increasing the activities of antioxidant enzymes such as GPx and SOD. These results suggest that H2S can play an important role in regulating the lipid and antioxidant metabolism. However, further studies are needed to elucidate the precise underlying mechanism of lipid metabolism and antioxidant activities of H2S.

## Conclusion

In conclusion, we proved that H2S can restore the liver from steatosis in obese mice. As shown in Figure 5, H2S mitigates the fatty liver most likely through (1) suppressing hepatic fat accumulation by down-regulation of FAS and up-regulation of CPT-1; (2) up-regulating the activities of antioxidant enzymes (GPx and SOD).

## Abbreviations

NAFLD: Nonalcoholic fatty liver disease
HFD: High fat diet
NFD: Normal fat diet
TG: Triglyceride
TC: Total cholesterol
FAS: Fatty acid synthase
CPT-1: Carnitine palmitoyltransferase-1
MDA: Malondialdehyde
SOD: Superoxide dismutase
GPx: Glutathione peroxidase (GPx)
